# Clinical spectrum and factors associated with Brucella epididymo-orchitis among male patients in a high-endemic region of Northwest China

**DOI:** 10.3389/fpubh.2026.1757014

**Published:** 2026-06-17

**Authors:** Bofei Liu, Guangtian Liu, Xue Han, Ruiqing Zhang, Jiangping Li, Xiaohong Wang

**Affiliations:** 1 Ningxia Medical University, Yinchuan, China; 2 Fourth People Hospital of Ningxia, Yinchuan, China; 3 General Hospital of Ningxia Medical University, Yinchuan, China

**Keywords:** associated factors, brucellosis, clinical features, endemic region, epididymo-orchitis

## Abstract

**Background and objectives:**

BEO is a clinically important but often underrecognized localized manifestation of brucellosis in male patients, particularly in high-endemic pastoral regions. However, large-scale data describing its clinical spectrum and associated factors remain limited. This study aimed to characterize the epidemiological, clinical, and laboratory features of BEO among hospitalized male brucellosis patients in Northwest China and to explore clinical and laboratory factors associated with BEO.

**Methods:**

This retrospective cross-sectional study was conducted at the regional infectious disease hospital of Ningxia Hui Autonomous Region from January 2021 to December 2023. Male inpatients aged 16 years or older with laboratory-confirmed brucellosis were included. Patients were classified into BEO and non-BEO groups. Demographic, clinical, and laboratory data were extracted from medical records. Univariable analyses were performed to compare the two groups, and multivariable logistic regression was used to identify factors associated with BEO.

**Results:**

A total of 1,188 hospitalized male brucellosis patients were included, of whom 72 had clinically recognized and imaging-confirmed BEO, accounting for 6.06% of the study population. In univariable analyses, fatigue, lumbago, positive blood culture, platelet count, white blood cell count, ESR, and symptom duration differed between the BEO and non-BEO groups. In the multivariable model, fatigue (OR = 0.43, 95% CI: 0.23–0.81, *p* = 0.008), lumbago (OR = 0.41, 95% CI: 0.21–0.82, *p* = 0.011), white blood cell count (OR = 1.13, 95% CI: 1.02–1.25, *p* = 0.019), and positive blood culture (OR = 1.77, 95% CI: 1.03–3.04, *p* = 0.038) remained associated with BEO. Platelet count, ESR, and symptom duration were not statistically significant after adjustment.

**Conclusion:**

In this study, BEO was identified in 6.06% of hospitalized male brucellosis patients, and was associated with lower frequencies of fatigue and lumbago, higher white blood cell count, and positive blood culture. These findings suggest that BEO may represent a distinct localized clinical phenotype rather than a simple extension of systemic or osteoarticular disease. The observed associations should not be interpreted as causal predictors. Prospective multicenter studies with standardized screening, detailed exposure assessment, and longitudinal follow-up are needed to clarify the true burden, mechanisms, and long-term outcomes of BEO.

## Introduction

Brucellosis is a globally prevalent zoonotic infection caused by bacteria of the *Brucella* genus. It poses a significant threat to both public health and agricultural economies, particularly in regions with extensive livestock farming. Worldwide, an estimated 5 to 12.5 million new human cases occur annually (1, 2). In China, the burden of brucellosis remains disproportionately high in the northern pastoral provinces, with a notable resurgence in recent years. National surveillance data indicate that the reported incidence increased from 3.25 per 100,000 in 2019 to 4.99 per 100,000 in 2023, and the number of reported cases rose from 45,046 to 70,439 during this period (3–5). Early diagnosis and timely intervention are critical to prevent long-term complications and reduce transmission (6–8). In this context, the Ningxia Hui Autonomous Region—an endemic area in northwestern China with dense sheep farming—is a focal point for targeted brucellosis control efforts (9, 10).

Although brucellosis is a systemic infection with the potential to affect multiple organ systems, genitourinary involvement is particularly significant in male patients. Epididymo-orchitis is one of the most common localized complications, reported in approximately 2.57% of cases (11). *Brucella* spp. can evade host immune defenses and breach the blood–testis barrier, resulting in testicular inflammation, pain, swelling, and in severe cases, infertility or chronic discomfort (12, 13). Diagnosis often relies on clinical signs in conjunction with serological or microbiological confirmation. However, due to non-specific manifestations, BEO is frequently misdiagnosed as viral orchitis, or even malignancy, leading to delayed treatment and unnecessary interventions (14–18). While several studies have reported individual cases or small series of BEO, particularly in Middle Eastern and Mediterranean countries, large-scale epidemiologic data from high-prevalence regions such as northwest China remain scarce (11, 14).

Despite growing awareness of BEO, key knowledge gaps persist. First, the prevalence and demographic profile of BEO in endemic pastoral settings, particularly within China, have not been systematically described. Second, few studies have attempted to identify factors independently associated with testicular involvement by comparing male brucellosis patients with and without BEO. Third, the clinical heterogeneity within BEO cases remains poorly understood. Specifically, it is unclear whether differences exist between patients with isolated testicular involvement and those with additional systemic organ involvement in terms of presentation, laboratory findings, and underlying pathophysiology.

To address these gaps, we conducted a retrospective cross-sectional study based on comprehensive inpatient data from the only regional infectious disease hospital in Ningxia. This hospital serves as the primary referral center for brucellosis diagnosis and management across the province. Our objectives were threefold: (1) to characterize the demographic, clinical, and laboratory features of BEO patients; (2) to identify clinical and laboratory factors associated with testicular involvement by comparing BEO and non-BEO groups. By providing region-specific, stratified insights, this study aims to improve early recognition, support individualized clinical decision-making, and contribute to targeted public health strategies in high-burden areas.

## Methods

### Study design and setting

This was a hospital-based, retrospective cross-sectional study conducted at the only regional infectious disease hospital in Ningxia Hui Autonomous Region, Northwest China—a high-endemic area for brucellosis (9, 10). The hospital serves as the central referral center for brucellosis patients throughout the region. The study included all male brucellosis inpatients aged ≥16 years who were first diagnosed with brucellosis during their index hospitalization at our center between January 2021 and December 2023. No repeat hospitalizations for the same patient were included in the analysis.

This study was approved by the institutional ethics committee. Because this was a retrospective analysis of anonymized clinical data and involved no additional patient intervention, the requirement for written informed consent was waived in accordance with local ethical regulations.

### Participants and case definition

Eligible participants were male brucellosis inpatients aged 16 years or older who met the diagnostic criteria for brucellosis, including a clear history of epidemiological exposure, characteristic clinical features, and confirmation by either a standard agglutination test (SAT) titer of ≥1:160 or a positive *Brucella* culture (3, 5, 6). To minimize selection bias and avoid missing subclinical cases, all male brucellosis patients routinely underwent bedside scrotal ultrasonography during hospitalization, regardless of whether they reported scrotal symptoms. BEO was diagnosed only when both clinical manifestations (e.g., testicular pain and/or swelling) and confirmatory scrotal ultrasonography findings were present. No BEO case was diagnosed based on clinical evaluation alone. BEO was defined as documented epididymal and/or testicular involvement supported by compatible clinical manifestations, such as testicular pain or swelling, together with scrotal ultrasonography findings. Patients were excluded if they had incomplete clinical or laboratory data or serious underlying comorbidities that could confound the evaluation of testicular involvement. Serious underlying comorbidities were defined as conditions that could substantially confound the evaluation of focal brucellosis involvement, including tuberculosis, ankylosing spondylitis, rheumatic or rheumatoid disease, infective endocarditis, and brucellar meningitis.

### Group classification

Participants were divided into two groups for analysis. The BEO group included patients with brucellosis and testicular involvement. The non-BEO group comprised male brucellosis patients without testicular involvement.

### Data collection and variable definitions

Diagnostic data for BEO classification were reviewed from the medical records, including clinical examination findings and imaging reports, and group assignment (BEO vs. non-BEO) was based on these documented diagnostic criteria. Researchers used standardized data collection forms to extract information from electronic medical records. Two independent investigators reviewed all documentation, with any discrepancies resolved through consultation by a third reviewer. The collected variables included age, educational background, and clinical symptoms such as fever, fatigue, joint pain, spinal symptoms, excessive sweating, and testicular pain/swelling.

Laboratory data collected at admission included hematological parameters (white blood cell count, platelet count, lymphocyte percentage), liver function indicators, lactate dehydrogenase, albumin, etc.

### Statistical analysis

All statistical analyses were performed using SPSS version 25.0 (IBM Corp., Armonk, NY, USA). Continuous variables were tested for normality test. Normally distributed variables were expressed as mean ± standard deviation (SD) and compared using the independent samples t-test; non-normally distributed variables were presented as median (interquartile range) and compared using the Mann–Whitney *U* test. Categorical variables were summarized as counts and percentages and compared using the chi-square test or Fisher’s exact test, as appropriate.

Descriptive statistical methods were employed to summarize demographic, clinical, and laboratory characteristics in the BEO group. Furthermore, risk factors associated with testicular involvement were identified by comparing the BEO and non-BEO groups. Variables with *p* < 0.05 in univariable analyses were considered for inclusion in the multivariable logistic regression model, with adjusted odds ratios (OR) and 95% confidence intervals (CI) reported. However, variables directly involved in the diagnostic definition of BEO, including testicular swelling/pain and scrotal ultrasonography findings, were excluded from the multivariable model to avoid circular reasoning. We conducted a sensitivity analysis by forcibly incorporating predefined clinically relevant covariates (age, disease stage, and standard agglutination test (SAT) titer) into the multivariate model regardless of their univariate significance; where disease stage was used to represent the overall stage and severity of the disease.

Missing data were not imputed. Available-case analysis was used for descriptive comparisons, and complete-case analysis was used for multivariable logistic regression. Missing data for key variables are summarized in Supplementary Table S1. In two-tailed tests, *p* values <0.05 were considered statistically significant.

## Results

### Baseline characteristics of the study population

All patients classified as BEO had documented both clinical manifestations and imaging-supported evidence of epididymo-orchitis; no BEO diagnosis was based on clinical examination alone (100% imaging-supported confirmation).

The final analysis included 1,188 first-hospitalized male patients with brucellosis, with an average age of 48.39 years. The majority (31.5%) had completed primary school education. The most common clinical manifestations were arthritis (89.1%), joint effusion (74.0%), and arthralgia (45.2%). The average body temperature was 37.24 °C°C. Detailed laboratory test results are presented in Table 1.

**Table 1:** Baseline characteristics of the study population.

Feature	Total (*n* = 1,188)
Age, years, mean (SD)	48.39 (14.40)
Education level	
Illiterate, *n* (%)	160 (13.5)
Primary school, *n* (%)	374 (31.5)
Junior high school, *n* (%)	296 (24.9)
High school/Technical secondary school, *n* (%)	302 (25.4)
College/University, *n* (%)	43 (3.6)
Graduate and above, *n* (%)	13 (1.1)
BMI, kg/m^2^, mean (SD)	23.08 (3.90)
Stage, *n* (%)	
Acute phase	991 (83.4)
Subacute phase	118 (9.9)
Chronic phase	78 (6.6)
Fatigue, *n* (%)	419 (35.3)
Arthralgia, *n* (%)	537 (45.2)
Hidrosis, *n* (%)	66 (5.6)
Myalgia, *n* (%)	22 (1.9)
Fever, *n* (%)	350 (29.5)
Lumbago, *n* (%)	325 (27.4)
Anorexia, *n* (%)	59 (5.0)
Splenomegaly, *n* (%)	271 (22.8)
Testicular swelling and pain, *n* (%)	37 (3.1)
Arthritis, *n* (%)	1,059 (89.1)
Spondylitis, *n* (%)	444 (37.4)
Paravertebral abscess, *n* (%)	92 (7.7)
Intraspinal abscess, *n* (%)	46 (3.9)
Hydrops articuli, *n* (%)	879 (74.0)
Blood culture, *n* (%)	
Lymphadenopathy, *n* (%)	78 (6.6)
Temperature, °C, Mean (SD)	37.24 (0.89)
PLT, ×10^9^/L, mean (SD)	212.32 (64.21)
ALT, U/L, mean (SD)	25.26 (27.10)
AST, U/L, mean (SD)	27.69 (34.27)
GGT, U/L, mean (SD)	51.60 (62.52)
ALB, g/L, mean (SD)	34.72 (4.28)
WBC, ×10^9^/L, mean (SD)	5.09 (1.99)
Lymphocytes, ×10^9^/L, mean (SD)	1.66 (0.65)
Total bilirubin, μmol/L, mean (SD)	11.67 (9.21)
LDH, U/L, mean (SD)	205.72 (101.07)
SAT, IU/mL, mean (SD)	423.92 (274.71)
Amylase, U/L, mean (SD)	71.10 (25.90)
PCT, ng/mL, mean (SD)	0.09 (0.08)
IL-6, pg./mL, mean (SD)	13.76 (25.45)
ESR, mm/h, mean (SD)	23.81 (22.52)

### Univariable analysis comparing the BEO and non-BEO groups

Among the 1,188 male patients with brucellosis, 72 were diagnosed with clinically recognized and imaging-confirmed BEO, corresponding to a proportion of 6.06%. In the univariable analysis, patients with BEO were less likely to report fatigue, lumbago than those without BEO. Positive blood culture was more frequent in the BEO group than in the non-BEO group. Regarding laboratory parameters, patients with BEO had higher platelet counts, higher white blood cell counts, and higher ESR levels. No significant between-group differences were observed in age, education level, disease stage, BMI, fever, arthralgia, brucellar spondylitis, paravertebral abscess, intraspinal abscess, hydrops articuli, lymphadenopathy, SAT titer, liver function parameters, albumin, lymphocyte count, PCT, IL-6, or other laboratory indicators (Table 2).

**Table 2:** Comparison between brucellosis with BEO and without BEO groups in univariate analysis.

Feature	BEO (*n* = 72)	non-BEO (*n* = 1,116)	*p*-value
Age, years, mean (SD)	46.72 (13.87)	48.50 (14.43)	0.311
Education level			0.151
Illiterate, *n* (%)	15 (20.8)	145 (13.0)	
Primary school, *n* (%)	14 (19.4)	360 (32.3)	
Junior high school, *n* (%)	20 (27.8)	276 (24.7)	
High school/Technical secondary school, *n* (%)	20 (27.8)	282 (25.3)	
College/University, *n* (%)	3 (4.2)	40 (3.6)	
Graduate and above, *n* (%)	0 (0.0)	13 (1.2)	
Stage, *n* (%)			0.667
Acute phase	61 (84.7)	930 (83.3)	
Subacute phase	8 (11.1)	110 (9.9)	
Chronic phase	3 (4.2)	75 (6.7)	
Symptom duration, per 30 days, mean (SD)	1.83 (2.58)	2.32 (3.98)	0.031
BMI, kg/m^2^, Mean (SD)	22.59 (3.82)	23.11 (3.91)	0.267
Fatigue, *n* (%)	15 (20.8)	404 (36.2)	0.008
Arthralgia, *n* (%)	26 (36.1)	511 (45.8)	0.110
Hidrosis, *n* (%)	1 (1.4)	65 (5.8)	0.184
Myalgia, *n* (%)	0 (0.0)	22 (2.0)	0.393
Fever, *n* (%)	27 (37.5)	323 (28.9)	0.123
Lumbago, *n* (%)	12 (16.7)	313 (28.0)	0.036
Anorexia, *n* (%)	2 (2.8)	57 (5.1)	0.547
Splenomegaly, *n* (%)	22 (30.6)	249 (22.3)	0.106
Arthritis, *n* (%)	65 (90.3)	994 (89.1)	0.749
Spondylitis, *n* (%)	22 (30.6)	422 (37.8)	0.217
Paravertebral abscess, *n* (%)	4 (5.6)	88 (7.9)	0.473
Intraspinal abscess, *n* (%)	2 (2.8)	44 (3.9)	0.856
Hydrops articuli, *n* (%)	51 (70.8)	828 (74.2)	0.529
Positive blood culture, *n* (%)	31 (43.1)	336 (30.1)	0.021
Lymphadenopathy, *n* (%)	7 (9.7)	71 (6.4)	0.384
Temperature, °C, Mean (SD)	37.36 (1.07)	37.24 (0.88)	0.354
PLT, ×10^9^/L, mean (SD)	233.13 (83.57)	211.01 (62.61)	0.034
ALT, U/L, mean (SD)	28.23 (30.56)	25.06 (26.87)	0.338
AST, U/L, mean (SD)	24.04 (20.57)	27.92 (34.96)	0.352
GGT, U/L, mean (SD)	53.91 (40.56)	51.45 (63.69)	0.746
ALB, g/L, mean (SD)	34.75 (4.63)	34.75 (4.26)	0.998
WBC, ×10^9^/L, mean (SD)	5.86 (3.39)	5.04 (1.86)	0.046
Lymphocytes, mean (SD)	1.72 (0.71)	1.65 (0.64)	0.392
Total bilirubin, μmol/L, mean (SD)	10.50 (5.71)	11.74 (9.39)	0.269
LDH, U/L, mean (SD)	194.56 (67.93)	206.44 (102.83)	0.366
SAT, mean (SD)	464.08 (269.28)	421.35 (274.98)	0.204
Amylase, U/L, mean (SD)	73.63 (24.16)	70.85 (26.12)	0.656
PCT, ng/mL, mean (SD)	0.09 (0.07)	0.09 (0.09)	0.786
IL-6, pg./mL, mean (SD)	22.79 (64.48)	13.07 (19.60)	0.469
ESR, mm/h, mean (SD)	31.40 (22.37)	23.32 (22.46)	0.004

### Multivariable analysis comparing the BEO and non-BEO groups

After adjustment those ariables with *p* < 0.05 in the univariable analysis, fatigue remained negatively associated with BEO. Compared with patients without fatigue, those with fatigue had lower odds of BEO (OR = 0.43, 95% CI: 0.23–0.81, *p* = 0.008). Similarly, lumbago was negatively associated with BEO (OR = 0.41, 95% CI: 0.21–0.82, *p* = 0.011). By contrast, white blood cell count was positively associated with BEO; each 1 × 10^9^/L increase in white blood cell count was associated with higher odds of BEO (OR = 1.13, 95% CI: 1.02–1.25, *p* = 0.019). Positive blood culture was also independently associated with BEO (OR = 1.77, 95% CI: 1.03–3.04, *p* = 0.038). Platelet count, ESR, and symptom duration were not statistically significant after adjustment (Table 3). Hosmer–Lemeshow goodness-of-fit test, *χ*^2^ = 21.98, df = 10, *p* = 0.079; discrimination (AUC) = 0.68; Nagelkerke *R*^2^ = 0.09. Multicollinearity assessed by variance inflation factor (VIF): all VIF < 1.3 (maximum VIF = 1.20), indicating negligible collinearity.

**Table 3:** Multivariate logistic regression analysis of predictors of BEO.

Feature	Reference group	OR (95% CI)	*p*-value
Fatigue	No	0.43 (0.23, 0.81)	0.008
Lumbago	No	0.41 (0.21, 0.82)	0.011
WBC		1.13 (1.02, 1.25)	0.019
PLT		1.00 (1.00, 1.01)	0.066
ESR		1.01 (1.00, 1.02)	0.132
Positive blood culture	No	1.77 (1.03, 3.04)	0.038
Symptom duration, per 30 days		1.00 (0.92, 1.08)	0.977

Model performance: Hosmer–Lemeshow goodness-of-fit test, *χ*^2^ = 21.98, df = 8, *p* = 0.079, discrimination (AUC) = 0.68; Nagelkerke *R*^2^ = 0.09. Multicollinearity assessed by variance inflation factor (VIF): all VIF < 1.3 (maximum VIF = 1.20), indicating negligible collinearity.

### Sensitivity analysis

In the sensitivity analysis additionally forcing in age, disease phase, and SAT titer (Table 4), the associations of fatigue (OR = 0.44, 95% CI: 0.23–0.81), lumbago (OR = 0.43, 95% CI: 0.22–0.86), and white blood cell count (OR = 1.13, 95% CI: 1.01–1.25) with BEO remained statistically significant, whereas the association with positive blood culture was attenuated and no longer statistically significant (OR = 1.70, 95% CI: 0.97–2.98, *p* = 0.063).

**Table 4:** Multivariate logistic regression sensitivity analysis of BEO-related factors.

Feature	Reference group	OR (95% CI)	*p*-value
Fatigue	No	0.44 (0.23, 0.81)	0.009
Lumbago	No	0.43 (0.22, 0.86)	0.017
WBC		1.13 (1.01, 1.25)	0.029
PLT		1.00 (1.00, 1.01)	0.068
ESR		1.01 (1.00, 1.02)	0.085
Positive blood culture	No	1.70 (0.97, 2.98)	0.063
Symptom duration, per 30 days		0.96 (0.78, 1.18)	0.669
Age		0.99 (0.97, 1.00)	0.125
Stage	Acute phase		
Subacute phase		1.82 (0.15, 21.50)	0.636
Chronic phase		2.04 (0.65, 6.42)	0.224
SAT		1.000 (0.999, 1.001)	0.500

## Discussion

This retrospective cross-sectional study characterized the clinical spectrum of BEO among hospitalized male patients with brucellosis in a high-endemic region of Northwest China and explored clinical and laboratory factors associated with BEO. Among 1,188 male brucellosis inpatients, 72 patients had clinically recognized and imaging-confirmed BEO, accounting for 6.06% of the study population. Fatigue, lumbago, WBC count, and positive blood culture remained associated with BEO, whereas platelet count, ESR, and symptom duration were not statistically significant after adjustment.

The main finding of this study is that BEO was characterized not only by localized genitourinary manifestations but also by a distinct pattern of non-diagnostic clinical and laboratory correlates. Patients with BEO were less likely to report fatigue and lumbago, but more likely to have higher WBC counts and positive blood culture. These findings suggest that BEO may not simply represent a manifestation of more extensive systemic or osteoarticular disease. Instead, it may present as a relatively localized form of brucellosis with concurrent evidence of inflammatory or microbiological activity.

A distinctive feature of the present study, compared with many previous reports that mainly described BEO through case reports or small case series, is that it evaluated BEO within a large hospitalized brucellosis cohort and directly compared patients with and without BEO. The negative associations of fatigue and lumbago with BEO are clinically noteworthy but should be interpreted cautiously. They should not be regarded as protective factors or causal determinants, but rather as cross-sectional associations that may reflect different symptom profiles, organ involvement patterns, diagnostic attention, or documentation practices. Overall, these findings highlight the clinical heterogeneity of BEO and support the need for integrated assessment of localized symptoms, imaging findings, and selected non-diagnostic clinical and laboratory features in endemic settings. We further emphasize that, given the retrospective single-center design, all of the observed associations—including those involving fatigue, lumbago, white blood cell count, and blood culture positivity—may reflect differences in clinical presentation patterns, referral and care-seeking pathways, and documentation practices rather than true underlying biological differences between patients with and without BEO. Accordingly, these associations should be regarded as hypothesis-generating signals to support diagnostic vigilance, not as established biological or causal relationships.

The clinical recognition of BEO as an important genitourinary complication of brucellosis has been well established, but its reported frequency and clinical spectrum vary substantially across regions and study populations. Previous studies have shown that epididymo-orchitis is one of the most common forms of genitourinary involvement in male brucellosis patients. In a retrospective study from Saudi Arabia, Memish and Venkatesh described 26 cases of BEO and emphasized that brucellosis should be considered in the differential diagnosis of epididymo-orchitis in endemic areas (19). In Turkey, Naz et al. (14) reported 21 patients with BEO and compared their clinical and laboratory features with those of brucellosis patients without BEO, highlighting that localized scrotal symptoms are central to clinical recognition. More recently, Celik et al. (11) conducted a multicenter study of testicular involvement in brucellosis and found that epididymo-orchitis was the most frequent form of testicular involvement, supporting the need to actively ask male brucellosis patients about scrotal pain and swelling. A Chinese retrospective study also summarized 69 cases of brucellar orchitis and showed that BEO may present with heterogeneous clinical manifestations rather than a single uniform phenotype (13). In the present cohort, clinically recognized and imaging-confirmed BEO accounted for 6.06% of hospitalized male brucellosis patients. This proportion is broadly consistent with the concept that BEO is an important but relatively uncommon localized manifestation. Differences in reported frequency across studies may be explained by variation in case definitions, ultrasound screening strategies, referral patterns, and whether the source population consisted of all brucellosis patients, hospitalized patients, or patients already suspected of testicular involvement.

The relationship between BEO and systemic symptoms remains incompletely defined and may differ according to disease localization and clinical evaluation pathways. Brucellosis is classically characterized by non-specific systemic manifestations, including fever, sweating, fatigue, arthralgia, and myalgia. A systematic review and meta-analysis of human brucellosis in China showed that fever, sweating, arthralgia, and fatigue were common clinical manifestations, reflecting the systemic nature of the disease (20). Regional data from Ningxia also demonstrated that hospitalized brucellosis patients frequently presented with systemic and osteoarticular symptoms, consistent with the high burden of musculoskeletal involvement in endemic settings (21). In contrast, studies focused on BEO have emphasized that localized scrotal pain and swelling may dominate the clinical presentation, sometimes leading patients to seek care through urological rather than infectious disease pathways (14, 19). In the present study, fatigue and lumbago were negatively associated with BEO after adjustment. This finding differs from the traditional expectation that localized complications necessarily occur in patients with more prominent systemic or musculoskeletal symptoms. It should not be interpreted as a protective effect of fatigue or lumbago. A more plausible explanation is that patients with BEO may present predominantly with localized genitourinary complaints, whereas patients without BEO may be more likely to have systemic or osteoarticular manifestations. In addition, retrospective symptom documentation may be influenced by the chief complaint and the clinician’s diagnostic focus at admission.

The association between BEO and inflammatory or microbiological activity has been reported in previous studies, but the specific laboratory markers associated with BEO have not been consistent. Akıncı et al. (22) compared brucellosis patients with and without epididymo-orchitis and found that positive blood culture was more frequent among patients with epididymo-orchitis, suggesting that detectable bacteremia may be linked to genitourinary involvement in some patients. Naz et al. (14) also compared BEO and non-BEO patients and reported differences in inflammatory and laboratory parameters, although the small sample size limited stable multivariable interpretation. In the multicenter study by Celik et al., the authors noted that blood culture remains an important diagnostic method and that laboratory abnormalities such as elevated inflammatory markers may be observed in different clinical forms of brucellosis (11). In the present study, positive blood culture and higher WBC count remained associated with BEO after adjustment. These findings may indicate that patients with BEO have more evident microbiological activity or inflammatory response at hospitalization. However, because all variables were measured during the same hospitalization period, these associations cannot establish temporal or causal relationships. They should instead be interpreted as clinically accessible correlates that may help maintain diagnostic vigilance when evaluating male brucellosis patients in endemic regions.

The role of conventional inflammatory markers and hematological indices in BEO remains controversial. Several previous studies have attempted to identify laboratory parameters that may help distinguish BEO from other forms of brucellosis or from non-brucellar epididymo-orchitis. Some reports suggested that CRP, ESR, NLR, MPV, RDW, or MLR may be useful auxiliary indicators for BEO or for differentiating brucellar from non-brucellar epididymo-orchitis (22–25). For example, studies comparing brucellar and non-brucellar epididymo-orchitis have explored the diagnostic value of inflammatory hematological indices, reflecting the clinical need for simple laboratory markers in endemic areas (23, 24). Nevertheless, these studies differed in comparator groups, endpoint definitions, timing of blood sampling, and whether the focus was BEO among brucellosis patients or differential diagnosis among epididymo-orchitis patients. In our study, PLT and ESR differed between BEO and non-BEO patients in univariable analysis but were no longer statistically significant after adjustment. This suggests that their crude associations may have been partly explained by other clinical or microbiological variables. Therefore, although inflammatory markers remain useful for assessing the general inflammatory status of brucellosis patients, their independent relationship with BEO appears unstable and should be interpreted cautiously.

Taken together, the present findings extend previous evidence in two ways. First, this study evaluated BEO within a relatively large hospitalized brucellosis cohort from a high-endemic region of Northwest China, rather than relying only on case reports or small case series. Second, by excluding case-defining symptoms from the revised multivariable model, the analysis focused on non-diagnostic clinical and laboratory correlates of BEO. This approach supports a more cautious interpretation: BEO should be recognized as a heterogeneous localized manifestation characterized by scrotal involvement, but its associated clinical profile may not simply reflect greater systemic disease burden. Future prospective studies with standardized ultrasound screening, detailed symptom chronology, microbiological assessment, and longitudinal follow-up are needed to clarify whether these observed associations reflect disease activity, organ tropism, referral pathways, or documentation patterns.

### Limitations

This study has several limitations. First, this was a single-center retrospective cross-sectional study conducted at a regional infectious disease hospital in a high-endemic area. Although the hospital serves as an important referral center for brucellosis in Ningxia, the study population consisted of hospitalized patients and may not fully represent all male brucellosis patients in the community or in primary care settings. This may have introduced referral and selection bias, particularly because patients admitted to a specialized hospital may have more complicated disease manifestations or different healthcare-seeking patterns. Therefore, the observed proportion of BEO and its associated clinical features should be interpreted in the context of hospitalized brucellosis patients rather than the general brucellosis population. Future multicenter studies including community-based, outpatient, and inpatient populations from different endemic regions are needed to improve the generalizability of these findings.

Second, because of the retrospective design, clinical information was extracted from existing medical records. Symptoms such as fatigue, lumbago, and scrotal discomfort may have been affected by incomplete documentation, differences in clinicians’ questioning, and patients’ chief complaints at admission. This limitation may have influenced the observed associations between symptom profiles and BEO, especially the negative associations of fatigue and lumbago. These findings should therefore be interpreted as clinical correlations rather than true protective effects or causal relationships. Future prospective studies should use standardized symptom assessment forms, predefined definitions, and uniform data collection procedures to reduce information bias. We further emphasize that, given the retrospective, single-center design of this study, all observed associations may reflect differences in clinical presentation patterns, referral and treatment pathways, and recording methods rather than genuine biological differences between BEO patients and non-BEO patients, nor do they represent established biological or causal relationships.

Third, the cross-sectional nature of the study precludes the establishment of temporal or causal relationships. Clinical symptoms, laboratory markers, blood culture results, and BEO status were evaluated during the same hospitalization period. As a result, it is not possible to determine whether factors such as higher WBC count or positive blood culture preceded, accompanied, or resulted from localized genitourinary involvement. Therefore, the multivariable findings should be interpreted as associations rather than causal predictors. Longitudinal cohort studies with repeated clinical, microbiological, and laboratory assessments are needed to clarify the temporal relationship between systemic disease activity and BEO. All observed associations may reflect differences in clinical presentation patterns, referral and treatment pathways, and recording methods, rather than genuine biological differences between BEO patients and non-BEO patients; these associations do not represent established biological or causal relationships.

Fourth, some potentially important confounding factors were unavailable in the database. Previous antibiotic exposure and detailed occupational exposure intensity were not systematically recorded and therefore could not be adjusted for. Prior antimicrobial therapy may reduce blood culture positivity and modify clinical manifestations, while occupational exposure intensity may influence bacterial inoculum, repeated exposure, and disease presentation. The absence of these variables may have resulted in residual confounding and may partly explain differences between this study and previous reports. Future prospective studies should collect detailed information on pre-admission antibiotic use, animal contact frequency, livestock-related tasks, protective measures, and duration of occupational exposure.

Fifth, although disease phase, symptom duration, blood culture positivity, and variables related to focal organ involvement were considered in the revised analysis, some aspects of disease heterogeneity could not be fully captured. For example, lumbago was used as a clinically available indicator related to possible osteoarticular or spinal involvement, but it cannot fully replace standardized imaging-confirmed assessment of focal lesions. Similarly, disease phase and symptom duration were based on clinical records and may not precisely reflect the biological stage of infection. Future studies should combine standardized clinical evaluation, imaging confirmation of focal organ involvement, and detailed chronology of symptom onset to better characterize the relationship between systemic and localized manifestations.

Finally, this study did not include treatment response or long-term outcomes. Therefore, we could not evaluate whether the identified associated factors were related to recurrence, chronic scrotal pain, fertility impairment, treatment failure, or other clinically important sequelae. This limits the ability to determine the prognostic significance of the observed clinical and laboratory differences. Future multicenter prospective cohorts with standardized treatment information and long-term follow-up are needed to evaluate the clinical consequences of BEO and to guide individualized management strategies in endemic regions.

## Conclusion

In this retrospective cross-sectional study from a high-endemic region of Northwest China, BEO was identified in 6.06% of hospitalized male brucellosis patients. After adjusting co-variables, BEO was associated with lower frequencies of fatigue and lumbago, higher WBC count, and positive blood culture, suggesting that BEO may represent a distinct localized clinical phenotype rather than a simple extension of systemic or osteoarticular disease. These findings have important clinical implications. For patients, improved recognition of BEO may help reduce diagnostic delay and prevent potential long-term sequelae. For clinicians, careful assessment of localized genitourinary symptoms, combined with scrotal ultrasonography, blood culture, and routine laboratory markers, may support earlier identification and individualized management. From a public health perspective, strengthening awareness of male genitourinary involvement in endemic regions may improve standardized diagnosis, clinical training, and targeted surveillance. Given the retrospective cross-sectional design, the observed associations should not be interpreted as causal predictors. Prospective multicenter studies with standardized screening, detailed exposure assessment, and longitudinal follow-up are needed to clarify the true burden, mechanisms, and long-term outcomes of BEO.

## Data Availability

The original contributions presented in the study are included in the article/Supplementary material, further inquiries can be directed to the corresponding author.
