# Supplementary material for: Clinical spectrum and factors associated with Brucella epididymo-orchitis among male patients in a high-endemic region of Northwest China
Source: Front Public Health. 2026 Jun 17;14:1757014. doi: 10.3389/fpubh.2026.1757014 (PMC13319086; doi:10.3389/fpubh.2026.1757014)
Supplement: Supplementary file 1 [file Data_Sheet_1.DOCX]

Table S1. Missing data listing

| **Variable** | **Missing n (%)** | **%** |
| --- | --- | --- |
| BEO diagnosis | 0 | 0.0 |
| Age | 0 | 0.0 |
| Education | 0 | 0.0 |
| Ethnicity | 0 | 0.0 |
| Occupation | 0 | 0.0 |
| Disease phase | 1 (0.1) | 0.1 |
| Arthralgia | 0 | 0.0 |
| Myalgia | 0 | 0.0 |
| Fever | 0 | 0.0 |
| Fatigue | 0 | 0.0 |
| Sweating | 0 | 0.0 |
| Lumbago | 0 | 0.0 |
| Anorexia | 0 | 0.0 |
| Testicular swelling/pain | 0 | 0.0 |
| SAT titer at admission | 7 (0.6) | 0.6 |
| WBC count | 0 | 0.0 |
| Lymphocyte count | 0 | 0.0 |
| Platelet count | 26 (2.2) | 2.2 |
| ESR | 26 (2.2) | 2.2 |
| ALT | 0 | 0.0 |
| AST | 0 | 0.0 |
| Albumin | 0 | 0.0 |
| Blood culture | 0 | 0.0 |
| Duration before admission | 2 (0.2) | 0.2 |
| Arthritis diagnosis | 0 | 0.0 |
| Spondylitis diagnosis | 0 | 0.0 |
| Paravertebral abscess | 0 | 0.0 |
| Intraspinal abscess | 0 | 0.0 |
| Splenomegaly | 0 | 0.0 |
| Joint effusion | 0 | 0.0 |
| Lymphadenopathy | 0 | 0.0 |

Missing percentages were calculated using the full analytical cohort (N = 1,188) as the denominator. Descriptive analyses used available data for each variable, and multivariable logistic regression was conducted using complete cases.
